# Beyond the counter: Navigating the landscape of Deanxit® dispensing – Insights from Jordanian community pharmacies

**DOI:** 10.1016/j.heliyon.2024.e28028

**Published:** 2024-03-15

**Authors:** Rawand E. Ahmad, Muna Barakat

**Affiliations:** Department of Clinical Pharmacy and Therapeutics, Faculty of Pharmacy, Applied Science Private University, Amman, 11931, Jordan

**Keywords:** Flupentixol, Melitracen, Deanxit, Substance use, Knowledge, Practice

## Abstract

**Background:**

This study investigates the dispensing patterns and knowledge of community pharmacists regarding Deanxit®, a combination of melitracen and flupentixol approved for the treatment of depression and anxiety in Jordan.

**Methods:**

This study employed a mixed-methods approach, involving 75 randomly selected pharmacies in two Jordanian governorates (Amman and Irbid). The investigation involved simulated patient scenarios and structured interviews employing a validated tool.

**Results:**

The analysis revealed that 70.6% of pharmacists were willing to dispense Deanxit® based on simulated scenarios, indicating malpractice. The mean practice score was 0.5867 out of 18, underlining a substantial level of malpractice. Pharmacists demonstrated poor knowledge, with a median score of 2.0 out of 15, reflecting a lack of awareness about Deanxit's labeled indications. Furthermore, 96% reported dispensing without prescriptions, and 62.1% acknowledged encountering cases of improper Deanxit® use.

**Conclusion:**

This study provides valuable insights into the current landscape of Deanxit® dispensing patterns, pharmacists' poor knowledge levels, and malpractices in Jordan. By identifying areas for improvement and offering recommendations for intervention, this study contributes to enhancing pharmacy practice and patient care outcomes in the region.

## 1 Introduction

Deanxit is a combined medication containing a tricyclic antidepressant (melitracen 10 mg) and an antipsychotic (flupentixol 0.5 mg) that marketed as an anxiolytic and antidepressant, leveraging the potential synergistic effects of these two drugs to enhance mood improvement [1]. As stated in the leaflet (Appendix 1), Deanxit® is only approved for treating depression and anxiety, with no off-label uses mentioned [1,2].

Although clinically approved for use as an antidepressant and anxiolytic, Deanxit® is increasingly utilized to address a range of conditions beyond its intended scope. These applications include the treatment of gastric reflux disease, inflammatory bowel syndrome (IBS), depression, tinnitus, somatic pain disorder, and other ailments [1,2].

Drugs are classified into many classes based on their prescription behaviors. The diagnosis nature of diseases and the safety profile of treatments are important factors in drug classification. Prescription medications are frequently used to treat severe diseases, whilst non-prescription medications are employed to treat minor disorders [3]. The United States Food and Drug Administration (USFDA) defines a prescription medication as a drug prescribed by a physician, purchased at a pharmacy, intended for use by one person, and regulated by the FDA [4]. Deanxit® is one of the prescription-only medications. Whereas non-prescribed medication is a drug that is available without a prescription and can be purchased from a pharmacy [4,5].

According to the World Health Organization (WHO), self-medication is defined as the self-administration of certain drugs by the patient to manage a recognized illness or symptom without prior medical experience, [6,7]. Self-medication behavior is quite common, encompassing not only the use of Over-the-Counter (OTC) pharmaceuticals but also the consumption of previously prescribed drugs that are taken without medical guidance [8]. The use of self-medication has several personal and societal advantages. In nations with overloaded health systems, where scheduling an appointment with a doctor may be difficult, it promotes rapid access to medicine that provides rapid relief to the patient [9]. However, self-medication is also associated with a number of hazards for the patient who uses it and, in certain situations, for the community. Montastruc et al. [8] enumerated several risks linked to self-medication, such as incorrect self-diagnosis, inappropriate therapy selection, serious adverse effects and contraindications, drug-drug interactions, inappropriate route of administration, and the risk of improper drug use or dependence [8]. This underscores its association with a measure of low quality of care.

According to Wazaify et al., pharmacists have observed the malpractice in community pharmacies related to Deanxit® dispensing and use [9,10]. Additionally, in 2022, Jordanians complained about the absence of Deanxit in pharmacies [11,12]. Therefore, our study addresses a gap in the existing literature by focusing on Deanxit®, a medication that has garnered limited attention in research despite its potential implications for patient care. Moreover, it is crucial to understand the dispensing patterns and knowledge surrounding this medication, especially given its potential use and impact on patient health. While the study was conducted in Jordan, the findings hold relevance beyond its geographic boundaries. Understanding dispensing patterns and pharmacists' knowledge regarding a medication like Deanxit® can provide insights applicable to other regions facing similar challenges or where the medication is prescribed. The knowledge gained from our study can inform pharmacy practice guidelines and interventions internationally, aiding in the optimization of medication management and patient care. This study investigates the dispensing patterns and knowledge of community pharmacists regarding Deanxit® using a mixed-method approach.

## 2 Methods

### 2.1 Study design and settings

**Fig. 1:**
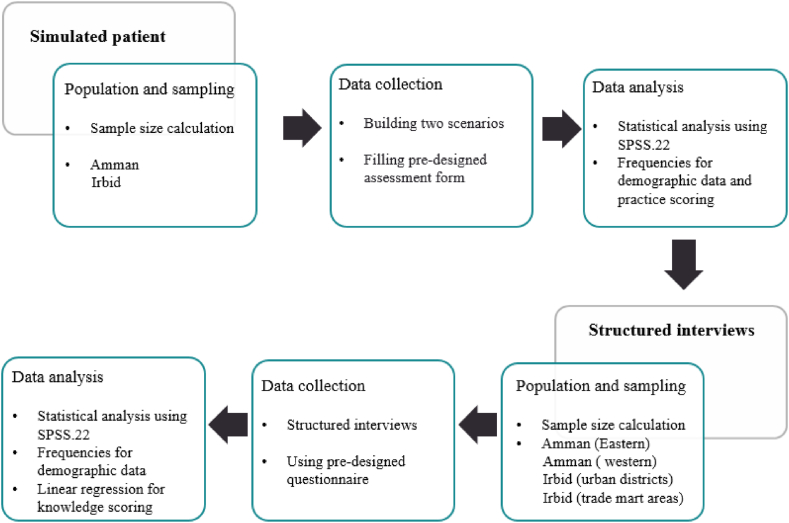
Decreptive scheme for the study mixed method.

This study is followed a mixed-method approach, as illustrated in Fig. 1. The observational study design involved the use of simulated patients (Mystery Shopper) and a structured face-to-face interview methodology. It was conducted at different community pharmacies in two main provinces in Jordan, Amman (the capital of Jordan) and Irbid (the second biggest province in terms of the population). The study was carried out from February 2023 to June 2023.

### 2.2 Ethical approval

Ethical approval to conduct the study was obtained from the Institutional Review Board (IRB) at Applied Science Private University (REF: 2022-PHA-40). Pharmacists who agreed to participate in the interviews provided verbal consent and were included in the study.

### 2.3 Phase 1: simulated patient

This study employed a simulated patient approach, utilizing a nontraditional observational design commonly referred to as “patient-actors.” These individuals closely resemble real patients and undergo training to enact specific scenarios, serving as a valuable tool for teaching and evaluation purposes [10]. The mystery shopper technique is unique because it directly observes events and behaviors in an uncontrolled environment. In our study, we used this method to evaluate pharmacists' behavior and dispensing patterns of Deanxit®. For the scope of the study, researchers included 75 different pharmacies (41 in Amman, 34 in Irbid) in the study. In each designated location, a convenience sample of community pharmacies was deliberately chosen for inclusion in the study. A proficient trained research assistant conducted visits to these selected pharmacies with the objective aim of obtaining securing signed consent forms from the responsible pharmacists.

Furthermore, a recruitment letter was provided to the pharmacist in charge. Additionally, all pharmacy staff members, elucidating were provided with a recruitment letter explaining that a simulated patient would be visiting their pharmacy seeking advice for alleviating symptoms of a specific certain illness. Those who expressed a desire not to participate were offered the option to wear a badge during the study period to signify their non-participation. Notably, specific details such as the scenario presented, the identity of the simulated patient, and the timing of the simulated visit were deliberately withheld from the participants to maintain the integrity of the study design. The simulated patient also adhered to the ethical guidelines established for this study to uphold the anonymity of participating individuals and safeguard the integrity of the acquired data. Initially, five pharmacies underwent were initially visited for the pilot study, to assess the validity and clarity. However, the, but their data from these pharmacies were subsequently were excluded from the final analysis [11].

The visits within each province were categorized into two distinct mystery shopper scenarios. The trained pharmacist assumed the role of a simulated patient in both scenarios during the visits. Immediately after each visit, the pharmacist completed a pre-designed assessment form (see Appendix 2). Adapted from previously published studies [12]. The form assembled information about pharmacy location, shift, pharmacist therapy approval based on provided scenarios, and any counseling points provided by the pharmacist during the pharmacy visit.

### 2.4 The used scenarios in the first phase

**Scenario A**: 40 community pharmacies distributed almost evenly between Amman n = 22 and Irbid n = 18 were selected and visited by a mystery shopper with irritable bowel syndrome. After clarifying the symptoms, the simulated patient showed the pharmacist the medication sachet claiming it was for his mother and asked if it was fine to take it? The scenario conducted was “*I have irritable bowel syndrome symptoms, including bloating, indigestion and abdominal pain, and my mother takes this medication for irritable bowel syndrome symptoms, is it okay if I take it too*?"

**Scenario B**: An additional 35 community pharmacies, evenly distributed between Amman (n = 19) and Irbid (n = 16), were selected and visited by the same simulated patient, this time portraying a scenario involving mood disturbances. After representing his symptoms, the simulated patient showed the pharmacist the medication sachet, claiming it was for his mother and asked if it was fine to take it too. The scenario conducted was " *I am in a bad mood due to a personal problem and I saw my mother take this medication whenever she is upset, is it okay if I take it too*?"

### 2.5 Practice scoring

The assessment criteria were established according to the patient care process for pharmacists as outlined by the Joint Commission of Pharmacy Practitioners [13]. Which is based on five primary disciplines, collect, assess, plan, implement and follow up, with subsections in each.

Participants were awarded one point for each correct answer, while zero points were assigned for incorrect or unknown responses. A total practice scores out of 18 was then calculated for each participant. Phase 2: Structured interview.

This phase was conducted after assessing the results of Phase 1. This method has been used to evaluate the pharmacists' knowledge and perception about Deanxit® using structured interviews with a pre-designed tool filled by the researcher (Appendix 3). It comprises a series of standardized, typically closed-ended questions administered to a fixed data sample in a standardized interview sequence [14].

### 2.6 Population and sampling (sample size calculation)

Amman was segmented into Eastern and Western areas, with the Eastern area reflecting a more traditional character, while the Western area, functioning as the city's economic center, adopts a more liberal atmosphere. In Irbid, the distribution encompassed urban districts and local trade mart areas.

Pharmacies were selected conveniently selected within the designated allocated areas, using a snowballing approach. Following the recommendations of Tabachnick and Fidell for sample size calculation in analysis, it is suggested that having 5–20 subjects per predictor is preferable [15]. Based on the number of independent variables levels used in this study (n = 11) and using the number of 20 subjects per predictor level, a minimum sample size of 220 or higher was considered suitable for the purpose of this study. The decision was made to increase the number to around 240 pharmacists to take into account missing responses and other unknown issues that might arise.

### 2.7 Validation and piloting of study tool

The study tool was developed and validated after a comprehensive review of related literature [[16], [17], [18], [19]]. Twenty pharmacists have assessed the content and face validity of the study tool which was prepared in English. According to their feedback, minor linguistic adjustments were implemented to improve the questionss clarity. The responses of the pilot study were not included in the final data analysis. The studytool consisted of three sections, the socio-demographic data, pharmacists' knowledge about Deanxit®, participants experience/practice with Deanxit® improper use. The time needed for the respondent to answer the questions ranged from 10 to 15 min.

### 2.8 Knowledge scoring

For the knowledge section, a series of questions regarding Deanxit® were asked. Each participant earned one point for each correct answer and zero point for each incorrect or do not know the answer. A total knowledge scores out of 18 was calculated for each participant. The median and IQR were calculated, and the cut-off point for good knowledge was 7.5 out of 15.

### 2.9 Statistical analysis

Data was analyzed using the Statistical Package for Social Sciences (SPSS) software version 22 (IBM Co. USA) to conduct statistical analyses. Data was presented as frequency (%), median ± interquartile range (IQR) and mean ± standard deviation (SD). Single linear regression was used to look for predictors that may affect knowledge scores. After performing univariate linear regression analysis, all variables having P-values <0.250 were put into multiple linear regression analysis. Furthermore, the multiple linear regression analysis identified variables that independently influenced participants' knowledge. Additionally, Chi-square test was used to determine if there was any significant difference between the independent variables, were P-values less than 0.05 was considered statistically significant.

## 3 Results

### 3.1 Phase 1: simulated patient phase

#### 3.1.1 Sociodemographic data

A total of 75 different community and chain pharmacies were visited by the simulated patient. Around half of the pharmacists were females (50.6%) and working in the mid-day shift B (53.4%). In parallel, more than two thirds of the visited pharmacies were individual pharmacies (76.0%). No significant differences were observed between the two conducted scenarios concerning the gender of the pharmacist included in the study, pharmacy location, pharmacy shift, or pharmacy status. Table 1 summarizes the characteristics and demographic data of the visited pharmacies.

**Table 1:** Sociodemographic Characteristics of the recruited pharmacies in both scenarios (n = 75).

Variable	First scenario: Irritable bowel syndrome scenario (n = 40)	Second Scenario: Mood disturbances scenario (n = 35)	P-value^a^
Gender of the pharmacist			
Female	17 (42.5)	21 (60.0)	0.134
Male	23 (57.5)	14 (40.0)	
**Pharmacy location**			
Amman	22 (55.0)	19 (54.2)	0.951
Irbid	18 (45.0)	16 (45.7)	
**Pharmacy shift**			
A	17 (42.5)	18 (51.4)	0.446
B	23 (57.5)	17 (48.5)	
**Pharmacy status**			
Chain	29 (72.5)	28 (80.0)	0.455
Individual	11 (27.5)	7 (20.0)	

a Chi-square test.

#### 3.1.2 Pharmacists agreement with Deanxit® administration

**Fig. 2:**
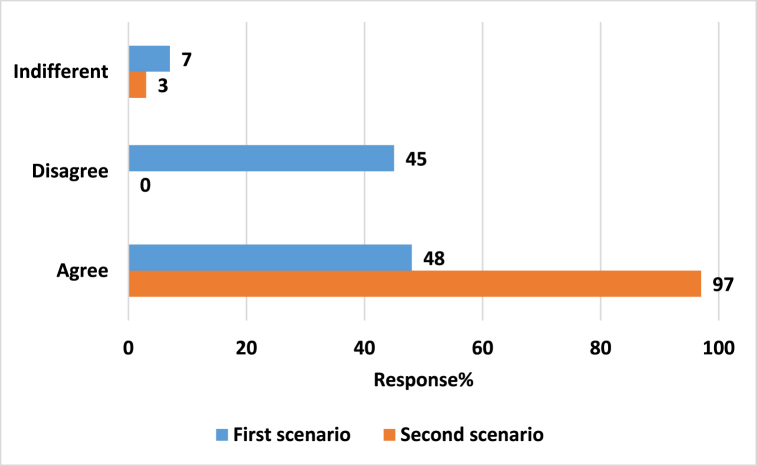
General Pharmacist's agreement with Deanxit® use according to mystery shopper scenarios.

As provided in Fig. 2, the majority of the pharmacists agreed with the use of Deanxit® in the first and second scenarios, 48% and 97%, respectively. Some of the pharmacists who disagreed with Deanxit's suggestion in the first scenario (IBS scenario), suggested an alternative. While, no alternatives were provided for the second (mood disturbances) scenario, as provided in (Fig. 1S), some of which are medications and others are herbal supplements. Poxidium**®** (Chlordiazepoxide and Clidinium bromide**)** was the most alternative prescribed (n = 8, 36.3%).

#### 3.1.3 Pharmacist's practice regarding Deanxit® dispensing

According to pharmacists' patient care process formulated by the joint commission of pharmacy practitioners, Table 2 shows that pharmacists’ mean practice score. As demonstrated, the pharmacy practice was poor, hence the score was 0.5867 out of 18 (SD = 0.68). Only 22 pharmacists out of 75 provided pharmaceutical education and instructions on how to use the drug (33.3%). While they provided a variable answer for Deanxit dosing Table 3.

**Table 2:** Pharmacists' practice and patient care process regarding Deanxit**®** in simulated patient scenarios (n = 75).

Pharmacist practice	n (%)
**Collect**	
Medication history	9 (12)
Medical history	0 (0)
Lifestyle habits	0 (0)
Previous use of the drug	6 (8)
**Assess**	
Medication appropriateness	0 (0)
Health and functional status	0 (0)
Immunization status	0 (0)
**Plan**	
Addresses medication related problems	4 (5.3)
Sets goals of therapy	0 (0)
Engages the patient through education	0 (0)
Support care continuity	0 (0)
**Implement**	
Initiates, modifies, discontinues, or administers medication therapy as authorize	0 (0)
Provides education and instructions on how to use the drug	25 (33.3)
Referral or transition of the patient to another health care professional	0 (0)
Schedules follow-up care	0 (0)
**Follow-up**	
Medication appropriateness, effectiveness, and safety and patient adherence	0 (0)
Clinical endpoints that contribute to the patient's overall health	0 (0)
Outcomes of care including progress toward goals of therapy	0 (0)

**Table 3:** Pharmacists’ responses regarding Deanxit® use (n = 25).

variable	n (%)
• One tablet at bedtime	5 (20%)
• One tablet twice a day	2 (8%)
• One tablet three times a day	4 (16%)
• One tablet three times a day for three days, then one tablet a day	1 (4%)
• One tablet PRN	6 (24%)
• Two tablets at bed time	3 (12%)
• Two tablets PRN	4 (16%)

### 3.2 Phase 2: structured interviews phase

#### 3.2.1 Sociodemographic characteristics of study participants

A total of 240 different community pharmacists were visited in this study. Eight pharmacists disagreed to take part in the study and five pharmacists did not recognize the medication (Deanxit®). Accordingly, 227 responses were included in the final analysis. Pharmacies were distributed in four different provinces, (n = 55, 24.2%) pharmacies in Eastern Amman, (n = 59, 26.0%) in Western Amman, (n-58, 25.6%) in urban districts in Irbid and (n = 55, 24.2%) in trade mart areas in Irbid. Among the responders, (n = 144, 63.4%) were females and most of the respondents held a bachelor's degree (n = 193, 85.0%). The sociodemographic characteristics of the respondents are presented in Table 4.

**Table 4:** Sociodemographic characteristics of the participant pharmacists (n = 227).

Variable	n (%)
Gender	
• Female	144 (63.4)
• Male	83 (36.6)
**Education level**	
• Diploma	22 (9.7)
• Bachelor's degree	193 (85.0)
• Postgraduates degree	12 (5.3)
• Pharmacy student + trainee	0 (0.0)
**Marital status**	
• Single	125 (56.8)
• Married	96 (45.8)
• Divorced/Widowed	6 (3.0)
**Years of experience**	
• <2	60 (26.5)
• 2-5	80 (35.2)
• >5	87 (38.3)
**Province of work**	
• Amman (Eastern)	55 (24.2)
• Amman (western)	59 (26.0)
• Irbid (urban districts)	58 (25.6)
• Irbid (trade mart areas)	55 (24.2)
**Pharmacy customers class distribution**	
• Low income	27 (11.9)
• Middle income	129 (56.8)
• Low-middle income	32 (14.0)
• High income	39 (17.3)

#### 3.2.2 Pharmacists’ experience with Deanxit**®** dispensing

**Fig. 3:**
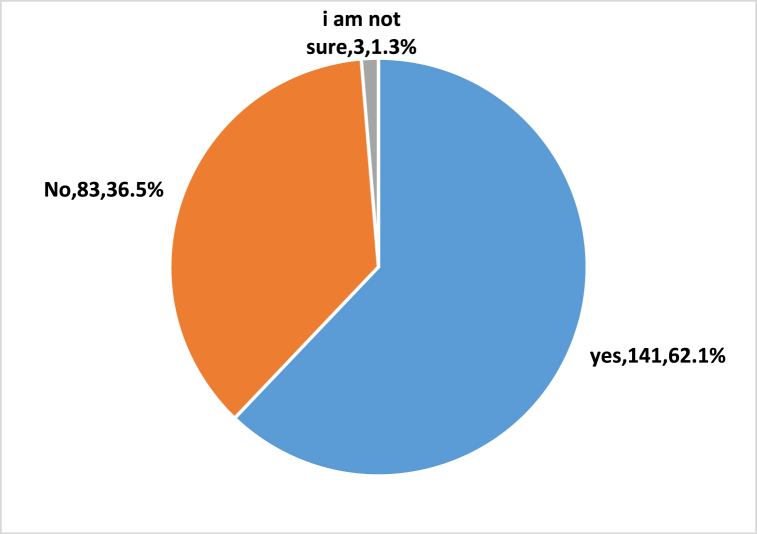
Pharmacists' reported exposure to improper use of Deanxit® according to structured interview (n = 227).

Although more than half of the interview pharmacists (n = 141,62.1%) reported the exposure to at least one case of improper use of Deanxit, Fig. 3. The majority of interviewed pharmacists (n = 217, 96%) reported that Deanxit used to be dispensed without a prescription.

Among the pharmacists who had ever been exposed to improper use of Deanxit® (n = 141), there were varied responses regarding its uses, including: " *Deanxit could be used for euphoria in an addictive way”* (n = 36, 25.5%) and *" Deanxit could be used for sleep disorders and insomnia*” (30, 21.2%). Other improper uses reported by interviewed pharmacists are listed in Table 1S.

Fig. 2S illustrates that the primary sources of information guiding improper users of Deanxit® are friends (n = 127) and family (n = 122), according to the pharmacists' responses in the structured interviews**.** Furthermore, the majority of pharmacists (n = 114, 80.8%) reported that improper users of Deanxit® frequently request it directly. Further data regarding improper users reported by the pharmacists are presented in Table 2S.

#### 3.2.3 Pharmacists practice toward patients who are suspected to use Deanxit® improperly

Eighty-four pharmacists (n = 84, 59.5%) declared that they would still dispense Deanxit®, even, in case of improper use confirmation. However, 43% of them stated the refusal to dispense or claim that the product is not available, more details in Table 3S.

#### 3.2.4 Pharmacists’ knowledge regarding Deanxit®

In general, pharmacists showed poor knowledge about Deanxit®. The overall median knowledge score was 2.0 out of 15 (IQR = 3.0). Aspects are all listed in Table 5. Upon asking if there are any unlabeled indications of Deanxit®, about three-quarters of pharmacists answered yes (n = 167, 73.5%), Table 4S lists these unlabeled indications that are mentioned by pharmacists in the interviews. Out of the 167 pharmacists who stated the presence of Deanxit® unlabeled indications, the most frequently mentioned indication was irritable bowel syndrome (n = 102, 61.0%), followed by sleep disorders (n = 60, 35.9%). Table 6 shows factors associated with pharmacists' knowledge score about Deanxit®, age, marital status, years of experience and highest education level were significantly affecting the knowledge score (P-value <0.05) at single linear regression. However, only the highest education level significantly affected the pharmacist knowledge score when multiple linear regression was conducted, in which the higher the education levels the highest the knowledge score.

**Table 5:** Pharmacists' knowledge regarding Deanxit® according to their answers in structured interviews (n = 227). More than one answer was allowed.

Variable	Answers n (%)
What are the labeled indications of Deanxit®	
• Anxiety (correct)	104 (45.8)
• Depression (correct)	87 (38.2)
• IBS (incorrect)	102 (44.9)
**Deanxit® has unlabeled indications**	
• Yes (incorrect)	167 (73.5)
• No (correct)	60 (26.4)
**Deanxit® side effects**	
• Insomnia, agitation (correct)	40 (17.6)
• Dizziness (correct)	157 (69.1)
• Ocular side effects (correct)	0 (0.0)
• Cardiac side effects (correct)	12 (5.2)
• Gastric side effects (correct)	14 (6.1)
**Deanxit® drug-drug interactions**	
• Monoamine oxidase inhibitors (correct)	11 (4.8)
• Adrenergic neuron blockers (correct)	4 (1.7)
• Anticholinergic agents (correct)	2 (0.8)
• Drugs that increase QT interval (correct)	14 (6.1)
• CNS depressants (correct)	136 (59.9)

Each correct answer was given 1-point, incorrect answer = zero point.

**Table 6:** Assessment of factors associated with pharmacists’ knowledge about Deanxit®.

Parameter	Knowledge score			
	Beta	P Value^a^	Beta	P Value^b^
**Age (years)**	Reference 0.185	**0.005**^c^	0.062	0.45
**Gender**	Reference	0.501	…	…
• **Female**	0.045			
• **Male**				
**The highest education level**	Reference	**0.001**^c^	0.22	**0.001**^d^
• **Diploma**	0.216			
• **Bachelor's degree**				
• **Postgraduates degree**				
• **Pharmacy student or trainer**				
**Marital status**	Reference	**0.003**^c^	0.095	0.187
• **Single**	0.197			
• **Married**				
• **Divorced/Widowed**				
**Years of experience**	Reference	**0.001**^c^	0.131	0.112
• **<2 years**	0.217			
• **2**–**5 years**				
• **>5 years**				
**Province where you work**	Reference	0.119	0.114	0.076
• **Amman (Eastern)**	0.101			
• **Amman (western)**				
• **Irbid (urban districts)**				
• **Irbid (trade mart areas)**				

a Using simple linear regression.

b Using multiple linear regression.

c Eligible for entry in multiple linear regression.

d Significant at 0.05 significance level.

## 4 Discussion

This study addresses a critical gap in the literature by presenting the first study on Deanxit® dispensing patterns among community pharmacists in Jordan. Deanxit® is a combination of a tricyclic antidepressant (TCA) and an atypical antipsychotic used in the treatment of anxiety and depression [20]. Despite being banned in several countries [21,22], it remains accessible in Jordan, raising concerns about its improper use. The study aims to evaluate pharmacists' knowledge and practices regarding Deanxit®, shedding light on the motivations behind its misuse and the current methods employed by pharmacists to manage such requests.

Deanxit’ s improper represents a serious health issue, a study on Deanxit® use disorder in Lebanon reported that 29.6% of participants used Deanxit® for purposes of relaxation and achieving a state of intoxication [19]. Our study showed that the practice score regarding Deanxit® dispensing was low, which indicates malpractice in pharmaceutical care delivery.

A minimal number of pharmacists inquired about the patient's medication history or previous use of the drug, which are standard procedures for patient data collection. This finding is consistent with a study conducted in Jordan examining the practice of pharmaceutical care in community pharmacies, where only 31.0% of pharmacists adhered to standard procedures for patient data collection [23]. The same study showed that 24.2% of the pharmacists documented over-the-counter recommendations which is consistent with our study were most pharmacists who carried out patient care provided education and instructions on how to use the drug (n = 25, 33.3%). According to Eman et al., this malpractice is hindered by barriers, mainly which are lack of supporting laws and poor therapeutic knowledge [23]. Another study showed that main barrier towards the provision of proper practice is the lack of training, which was noticed by about 80 % of the pharmacists in the study [24].

Furthermore, our study showed a wide variation in pharmacists' advice regarding counseling the simulated patient on how to take Deanxit®, which indicates the pharmacist's lack of knowledge of the medication dosing. Some pharmacists indicated that it should be taken as needed (PRN); however, to the best of the researcher's knowledge, no studies were discovered that investigated the effects of Deanxit® over a period of less than two weeks. Some pharmacists recommended a daily dose of three tablets; however, the leaflet indicates that the maximum dose is four tablets per day, with the maintenance dose being one tablet daily. A study about toxicological exposures supports this report on a telephonic consultation service at a tertiary care hospital in Lebanon. Deanxit® toxicity was reported in 2.7% of the cases, indicating the potential for severe toxicity in overdose [25].

In parallel, the study revealed a significant proportion of pharmacists who agreed with the use of Deanxit® in both scenarios presented to them. This indicates a general acceptance of Deanxit® as a treatment option for the simulated symptoms, suggesting that it is perceived as a viable therapeutic option among Jordanian pharmacists. Furthermore, the absence of significant differences between scenarios in terms of pharmacist characteristics and pharmacy settings implies a consistent trend in pharmacists' attitudes towards Deanxit® across different contexts. However, despite the agreement with Deanxit® administration, the study uncovered shortcomings in pharmacists' practice, particularly in providing pharmaceutical education and instructions on Deanxit® usage. This discrepancy between agreement and practice underscores the need for interventions aimed at bridging the gap between pharmacists' theoretical knowledge and their practical application in patient care settings. Improving pharmacists' ability to provide comprehensive patient counseling on medication usage and potential side effects is crucial for ensuring safe and effective medication management [26]. Unfortunately, in Jordan, drug dispensing laws and restrictions are not properly followed, and many medications—aside from opioids and controlled substances—are supplied in community pharmacies without a prescription [27]. This approach, which is mostly comparable to that of many other nations in the region, places more responsibility on community pharmacists to guarantee the safe and efficient use of pharmaceuticals and to assess whether seeking medical counsel is necessary [28].

In this study, based on observations of wrong dispensing patterns noticed by pharmacists in simulated patient scenarios, structured interviews were conducted to measure pharmacists' knowledge to determine the reason for pharmacists' dispensing patterns. The study revealed deficiencies in pharmacists' knowledge regarding Deanxit®. While a substantial proportion of pharmacists recognized unlabeled indications for Deanxit®, there were notable gaps in their overall knowledge scores. Similarly, research conducted in 2023 assessed community pharmacists' knowledge about psychiatric drugs, only 20.0% of 676 pharmacists completed the survey, and passed the minimum score (75.0%) for the knowledge questions concerning psychological medication [18]. Moreover, factors such as education level were found to significantly influence pharmacists' knowledge scores, highlighting the importance of continuous education and training programs for pharmacists to ensure they are equipped with accurate and updated information on medication usage and indications [29].

Of particular concern is the prevalence of improper Deanxit® use reported by pharmacists, including misuse for mood disorders, euphoria, and sleep disorders. Despite this, a significant proportion of pharmacists indicated they would still dispense Deanxit® even in cases of suspected improper use. This raises questions about pharmacists' role in mitigating potential harm associated with medication misuse and underscores the importance of implementing strategies to improve pharmacists' ability to identify and intervene in cases of inappropriate medication use [19].

Regarding the use of Deanxit® for irritable bowel syndrome was also reported by pharmacists, few studies in the literature mentioned Deanxit® use for IBS, a study assessing flupentixol and melitracen effect combined with trimebutine in patients with IBS accompanied with anxiety and depression showed that The incidence of improvement of gastrointestinal symptoms in the combined therapy group was 87.5% four weeks after treatment, which was greater than the rate in the control group [30].

Overall, these findings have significant implications for pharmacy practice and patient care in Jordan. Addressing the identified gaps in pharmacists' knowledge and practice regarding Deanxit® dispensing and usage is essential for enhancing patient safety and optimizing medication management. Targeted interventions, such as continuing education programs and practice guidelines, should be developed to equip pharmacists with the necessary skills and knowledge to provide optimal pharmaceutical care. Furthermore, collaboration between healthcare stakeholders, including pharmacists, prescribers, and policymakers, is crucial for implementing comprehensive strategies to address the challenges identified in this study and improve medication management practices in Jordan.

### 4.1 Study limitations

TheT study was conducted solely in Amman and Irbid, excluding other regions of the country due to transportation constraints. However, these are the two main provenience in Jordan with the highest population and registered pharmacies. Samples were collected during both Shift A (8:00am–4:00pm) and Shift B (4:00pm–12:00am); however, Shift C (12:00am–8:00am) was not included in the study.

This exclusion was due to cultural limitations, as a female researcher was responsible for sample collection during these hours. Additionally, simulated patient interviews were not recorded as approval for recording was not obtained. Moreover, self-reporting may be subject to inaccuracies, as pharmacists may be hesitant to disclose behaviors perceived as inappropriate by others. Furthermore, follow-up in practice scoring was not feasible, as the researcher would not revisit the same pharmacy. Consequently, progress toward therapy goals and clinical endpoints may not be established.

## 5 Conclusion

In conclusion, this study provides valuable insights into the current landscape of Deanxit® dispensing patterns, pharmacists' knowledge levels, and practices in Jordan. Pharmacists, presented a lack of proper education and knowledge regarding medication. Also, malpractice regarding Deanxit® dispensing was reported among pharmacists, although a number of participating pharmacists revealed the unwillingness to despise Deanxit® in case of improper use, their actual practice was found to be the opposite. Also, Deanxit® improper use was identified among the Jordanian population who have easy access to Deanxit® through community pharmacies without a prescription. Further studies are required to understand the areas for improvement and offering recommendations for intervention, this study contributes to enhancing pharmacy practice and patient care outcomes in the region.

## Consent to participate

All participants have provided a written informed consent electronically.

## Data availability statement

The dataset generated and analyzed in this study is available from the corresponding author on a reasonable request.

## CRediT authorship contribution statement

**Rawand E. Ahmad:** Writing – review & editing, Writing – original draft, Visualization, Project administration, Methodology, Formal analysis, Data curation, Conceptualization. **Muna Barakat:** Writing – review & editing, Writing – original draft, Visualization, Validation, Supervision, Software, Resources, Project administration, Methodology, Investigation, Funding acquisition, Formal analysis, Conceptualization.

## Declaration of competing interest

The authors declare that they have no known competing financial interests or personal relationships that could have appeared to influence the work reported in this paper.

## Footnotes

Appendix A Supplementary data to this article can be found online at https://doi.org/10.1016/j.heliyon.2024.e28028.
