# Supplementary material for: Beyond the counter: Navigating the landscape of Deanxit® dispensing – Insights from Jordanian community pharmacies
Source: Heliyon. 2024 Mar 15;10(6):e28028. doi: 10.1016/j.heliyon.2024.e28028 (PMC10966581; doi:10.1016/j.heliyon.2024.e28028)
Supplement: Multimedia component 1 [file mmc1.docx]

**Figure 1S.** Pharmacists' suggested alternatives other than Deanxit**®** in IBS scenario (n=22)

**Table 1S.** Improper uses of Deanxit® as reported by the participating pharmacists in the structured interviews (n=141).

| **Variable** | **n(%)** |
| --- | --- |
| For sleep disorders and insomnia | 30(21.2) |
| For pre-exams and interview tension | 9(6.3) |
| For mood disorders and to calm down | 37(26.2) |
| For premenstrual symptoms | 12(8.5) |
| For irritable bowel syndrome | 15(10.6) |
| For euphoria in an addictive way | 36(25.5) |
| For jaw muscles spasm during sleep | 1(0.7) |
| For increasing libido | 1(0.7) |

**Figure 2S.** The source of information that guides the Deanxit® improper user to use it according to pharmacists' responses in the structured interviews (n=141). More than one answer was allowed.

**Table 2S.** Characteristics of people who are vulnerable to Deanxit® improper use according to the reports of the pharmacists (n=141). More than one answer was allowed.

| **variable** | **n(%)** |
| --- | --- |
| **Gender** |  |
| - Female | 64(45.3) |
| - Male - Both | 21(24.8)  56(39.7) |
| **Age (years)** |  |
| - 20-30 | 26(18.4) |
| - 31-40 | 44(31.2) |
| - 41-50 | 69(48.9) |
| - >50 | 2(1.4) |
| **Visitors group** |  |
| - Strangers | 31(21.9) |
| - Regular pharmacy visitors | 38(26.9) |
| - Mix of both strangers and regular visitors | 72(51.0) |
| **How they are recognized** |  |
| - They ask directly for the drug | 114(80.8) |
| - Unusual appearance | 23(16.3) |
| - Mood disturbances | 2(1.4) |
| - Exaggerates symptoms to get medication | 14(9.9) |
| - Orders medication specifically and refuses alternatives | 18(12.7) |
| - Unwilling to provide reference information | 0(0.0) |
| - May show extraordinary knowledge of Deanxit® | 16(11.3) |
| - I can't recognize them | 0(0.0) |

**Table 3S.** The methods used by pharmacists to limit Deanxit® misuse and abuse (n=141).

| **Variable** | **n(%)** |
| --- | --- |
| - Refusal to dispense or claim that the product is not available | 61(43.2) |
| - Advise and clarify the side effects of these pills | 11(7.8) |
| - Requesting a prescription | 13(9.2) |
| - Hiding products from the shelf | 34(24.1) |
| - Working with JPA to solve the problem | 0(0.0) |
| - Referring the patient to the physician | 0(0.0) |
| - Report the improper use cases for the JFDA | 0(0.0) |
| - Conduct awareness campaigns | 0(0.0) |
| - Calling police | 1(0.7) |
| - Do nothing | 78(55.3) |

**Table 4S.** The unlabeled indications of Deanxit® according to the pharmacist who answered that Deanxit® has unlabeled indications (n=167). More than one answer was allowed.

| **Variable** | **n(%)** |
| --- | --- |
| irritable bowel syndrome | 102(61.0) |
| Migraine | 20(11.9) |
| Sleep disorders | 60(35.9) |
| Tinnitus | 1(0.5) |
| Anxiety | 16(9.5) |
| Depression | 15(8.9) |
| For mood disorders to calm down | 10(5.9) |
| Period agitation | 5(2.9) |
| Relax before exams and interviews | 2(1.1) |
