# Supplementary material for: Beyond the counter: Navigating the landscape of Deanxit® dispensing – Insights from Jordanian community pharmacies
Source: Heliyon. 2024 Mar 15;10(6):e28028. doi: 10.1016/j.heliyon.2024.e28028 (PMC10966581; doi:10.1016/j.heliyon.2024.e28028)
Supplement: Multimedia component 2 [file mmc2.pdf]

Simulated patient check list Pharmacist and pharmacy Information

Gender of the pharmacist	□ Female	□ Male
Pharmacy status	□ Individual	□ Chain
Pharmacy location	□ Amman	□ Irbid
Shift	□ Morning	□ Night
Did the pharmacist agree with the use of Deanxit, according to the case	□ Yes	□ No
Did the pharmacist recommended an alternative		
Notes		

Pharmacists' practice points and patient care process regarding Deanxit measured

**Collect**	**Yes**	**No**
○ Medication history		
○ Medical history		
○ Lifestyle habits		
○ Previous use of the drug		
**Assess**		
○ Medication appropriateness		
○ Health and functional status		
○ Immunization status		
**Plan**		
○ Addresses medication related problems		
○ Sets goals of therapy		
○ Engages the patient through education		
○ Support care continuity		
**Implement**		
○ Initiates, modifies, discontinues, or administers medication therapy as authorize		
○ Provides education and instructions on how to use the drug		
○ Referral or transition of the patient to another health care professional		
○ Schedules follow-up care		
**Follow-up**		
○ Medication appropriateness, effectiveness, and safety and patient adherence		
○ Clinical endpoints that contribute to the patient's overall health		
○ Outcomes of care including progress toward goals of therapy		
