# Supplementary material for: Beyond the counter: Navigating the landscape of Deanxit® dispensing – Insights from Jordanian community pharmacies
Source: Heliyon. 2024 Mar 15;10(6):e28028. doi: 10.1016/j.heliyon.2024.e28028 (PMC10966581; doi:10.1016/j.heliyon.2024.e28028)
Supplement: Multimedia component 3 [file mmc3.pdf]

Pharmacist knowledge and practice toward Deanxit® in Jordan

- **Your participation in this questionnaire is highly appreciated.**

- ○ Agree to participate
- ○ Disagree to participate

Part 1. Demographic data

- **Gender**

- ○ Female
- ○ Male

- **Age** ………………………………years

- **The highest education levels**

- ○ Diploma
- ○ Bachelor's degree
- ○ Postgraduates degree
- ○ Pharmacy student +trainer in a pharmacy

- **Marital status**

- ○ Single
- ○ Married
- ○ Divorced/Widowed

- **Years of experience**

- ○ <2
- ○ 2-5
- ○ >5

- **Province where you work**

- Amman (Eastern)
- Amman (western)
- Irbid (urban districts)
- Irbid (trade mart areas)

- **In general, the most common social class distribution of the pharmacy customers is (You can choose more than one option)**

- Low income
- Middle income
- Low-middle income
- High income

Part 2. Knowledge and beliefs of pharmacist about Deanxit uses

- **According to your knowledge, what are the labelled uses of Deanxit?**

	yes	no	Not sure
anxiety			
depression			
IBS			

- **According to your knowledge, Does Deanxit has unlabeled uses (i.e., a medicine prescribed for a purpose other than that for which it has been specifically designed and approved).**

- Yes
- No
- Not sure

- **If your answer was yes in the previous question, please specify**

	Yes	No	Not sure
Irritable bowel syndrome			
Migraine			
Sleep disorders			
Tinnitus			
Others			
Anxiety			
Depression			
For mood disorders to calm down			

Period agitation			
Relax before exams and interviews			

- **What are the possible side effects of Deanxit?**

	True	False
Psychiatric disorders (insomnia, agitation)		
Nervous system disorders (tremor , dizziness)		
Eye disorders		
Cardiac disorders		
Gastric disorders		
Others		

If you answered yes in the previous question, please proceed with the next question

- **What are the possible drug-drug interactions of Deanxit?**

	True	False
Monoamine oxidase inhibitors (MAOIs)		
Adrenergic neuron blockers like methyldopa		
Anticholinergic agents		
Drugs which may increase the QT interval		
CNS depressants:\		
Others		

Part 3. A. Pharmacist experience and practice towards Deanxit's improper use

- **According to experience, which is more common?**

  - Dispensing Deanxit with prescription
  - Dispensing Deanxit without prescription (as OTC)

- **Had you ever been exposed to any improper use cases of Deanxit?**

  - Yes
  - No
  - I am not sure

If you answered “Yes” in the previous question, please answer the following questions

- **What were those improper uses among the users, please mention them**

  …………………………………………………………………………………

- **Which gender group is/are more vulnerable to improper use?**

  - Males
  - Females
  - Both males and females

- **Which age group is/are more vulnerable to improper use?**

  - <20
  - 20-30
  - 31-40
  - 41-50
  - >50

- **Which group is/are more vulnerable to improper use?**

  - Strangers
  - Regular (known) pharmacy visitors
  - A mix of both strangers and visitors

- **What is the source of information that guides the Deanxit improper user to use it? (you can choose more than one option)**

  - Friend
  - Social media
  - Family
  - Pharmacist
  - Physician

3.B. Pharmacist practice towards Deanxit improper use

- **In case of confirmation of Deanxit improper use from certain pharmacy visitors, would dispense it for them?**

- Yes
- No

- **How do you usually recognize the improper use of Deanxit? (you can choose more than one option)**

  - They ask directly and acknowledge their needs
  - Unusual appearance (extreme lack of concern for appearance or dress)
  - Mood disturbances, inability to control impulses, and thought disturbances may appear
  - Exaggerates medical problems and/or mimics symptoms to get medication
  - Often orders medication specifically and refuses alternatives to other drugs
  - Reluctance or unwilling to provide reference information
  - May show extraordinary knowledge of Deanxit
  - I can't recognize them

- **What are the methods used by pharmacists to limit Deanxit misuse and abuse? (You can choose more than one option)**

  - Refusal to dispense or claim that the product is not available
  - Advise and clarify the side effects of these pills
  - Requesting a prescription
  - Hiding product from the shelf
  - Referring patient to the physician
  - Working with JPA to solve the problem
  - Report the improper use cases for the pharmacovigilance department of JFDA
  - Conduct awareness campaigns and pamphlets to raise awareness
  - Calling police
  - Do nothing
